# A Photovoltaic Self‐Powered Gas Sensor Based on All‐Dry Transferred MoS_2_/GaSe Heterojunction for ppb‐Level NO_2_ Sensing at Room Temperature

**DOI:** 10.1002/advs.202100472

**Published:** 2021-05-24

**Authors:** Yue Niu, Junwei Zeng, Xiangcheng Liu, Jialong Li, Quan Wang, Hao Li, Nicolaas Frans de Rooij, Yao Wang, Guofu Zhou

**Affiliations:** ^1^ Guangdong Provincial Key Laboratory of Optical Information Materials and Technology Institute of Electronic Paper Displays South China Academy of Advanced Optoelectronics South China Normal University Guangzhou 510006 P. R. China; ^2^ National Center for International Research on Green Optoelectronics South China Normal University Guangzhou 510006 P. R. China

**Keywords:** 2D materials, all‐dry transfer, gas sensing, heterojunction, photovoltaic, self‐powered

## Abstract

Traditional gas sensors are facing the challenge of low power consumption for future application in smart phones and wireless sensor platforms. To solve this problem, self‐powered gas sensors are rapidly developed in recent years. However, all reported self‐powered gas sensors are suffering from high limit of detection (LOD) toward NO_2_ gas. In this work, a photovoltaic self‐powered NO_2_ gas sensor based on n‐MoS_2_/p‐GaSe heterojunction is successfully prepared by mechanical exfoliation and all‐dry transfer method. Under 405 nm visible light illumination, the fabricated photovoltaic self‐powered gas sensors show a significant response toward ppb‐level NO_2_ with short response and recovery time and high selectivity at room temperature (25 °C). It is worth mentioning that the LOD toward NO_2_ of this device is 20 ppb, which is the lowest of the reported self‐powered room‐temperature gas sensors so far. The discussed devices can be used as building blocks to fabricate more functional Internet of things devices.

## Graphical abstract

A photovoltaic self‐powered gas sensor based on MoS_2_/GaSe heterojunction is fabricated via mechanical exfoliation and all‐dry transfer method. Under visible light illumination, large numbers of free carriers (electrons and holes) are produced and separated in the heterojunction, thus the MoS_2_/GaSe heterojunction can achieve self‐powered gas sensing toward ppb‐level NO_2_ at room temperature.

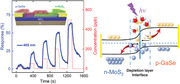

## 1 Introduction

Gas sensing technologies have recently attracted much attention owing to the significantly increasing threats of both industrial chemicals and daily air pollution.^[^ ^1^, ^2^ ^]^ As an important part of Internet of things devices, a new generation of gas sensor requires low power consumption and reliable selectivity, ensuring potential applications in smart phones and wireless sensor platforms.^[^ ^2^, ^3^ ^]^ Recent works have shown promising concepts to realize self‐powered gas sensors that are capable to detect gases dispensing the need for external electric power.^[^ ^4^ ^]^ Until now, three kinds of self‐powered gas sensors have been reported, which are based on piezoelectric, triboelectric, and photovoltaic effect, respectively.^[^ ^5^, ^6^, ^7^ ^]^ Especially, photovoltaic self‐powered gas sensors are activated by solar energy instead of mechanical power, which averts the demand of the complexity and heavy weight of vibrating devices.^[^ ^8^, ^9^ ^]^

The photovoltaic self‐powered gas sensors are normally fabricated with p‐n junctions, which could generate and separate carriers under light illumination. For example, Hoffmann et al. reported self‐powered gas sensors based on n‐ZnO/p‐Si.^[^ ^9^ ^]^ Since the band gap of ZnO is quite large (3.37 eV), only UV light could be used to activate the devices. In recent years, 2D materials have become promising alternatives to the metal oxide materials in room‐temperature gas sensing, which benefits from their inherent large surface‐to‐volume ratio and high carrier mobility.^[^ ^10^, ^11^ ^]^ Furthermore, 2D materials heterojunctions with photovoltaic properties have been used as current rectifiers, photodetectors, and solar cells.^[^ ^12^ ^]^ Nevertheless, 2D materials heterojunctions based photovoltaic self‐powered gas sensors are still barely reported. In 2020, Kim et al. reported 2D materials heterojunctions (MoS_2_/WSe_2_ and WSe_2_/WS_2_) based photovoltaic self‐powered gas sensors for the first time and explored their response to NO_2_ and NH_3_ with limit of detection (LOD) of 10 ppm.^[^ ^13^ ^]^ Note that compared with traditional 2D materials‐based gas sensors, all the reported self‐powered sensors are still suffering from higher LOD above 1 ppm.^[^ ^7^, ^9^, ^14^ ^]^ However, severe damages to human's eyes or respiration system will be inflicted once the NO_2_ concentration reaches only 1 ppm.^[^ ^15^ ^]^ As a member of the 2D materials family, GaSe, a layered metal‐monochalcogenide III‐VI semiconductor, shows excellent electrical and optical properties, high photoresponsivity and large specific surface area, thus making it a great candidate material as gas sensors. In 2020, Wu et al. reported a GaSe‐based NO_2_ gas sensor at room temperature with the LOD of 0.5 ppb.^[^ ^16^ ^]^ Wang et al. fabricated a self‐powered photodetector based on GaSe/WS_2_ heterojunction which showed high photoresponsivity and quite short response time.^[^ ^17^ ^]^ To the best of our knowledge, the heterojunction combining GaSe with other 2D materials as gas sensor has not been reported so far.

In this work, a photovoltaic self‐powered NO_2_ gas sensor based on the vertical MoS_2_ (n‐type)/GaSe (p‐type) heterojunction has been fabricated via mechanical exfoliation and all‐dry transfer method. A significant type II p‐n junction at the interface was expected to effectively modulate the carrier transport between the two materials. Under 405 nm light illumination, the MoS_2_/GaSe heterojunction exhibited excellent gas sensing performances toward NO_2_, including short response and recovery time and high selectivity both with bias voltage and in self‐powered mode. In particular, the LOD toward NO_2_ of the obtained photovoltaic self‐powered gas sensor reaches 20 ppb, which is the lowest among the reported self‐powered room‐temperature gas sensors so far.

## 2 Result and Discussion

**Figure 1:**
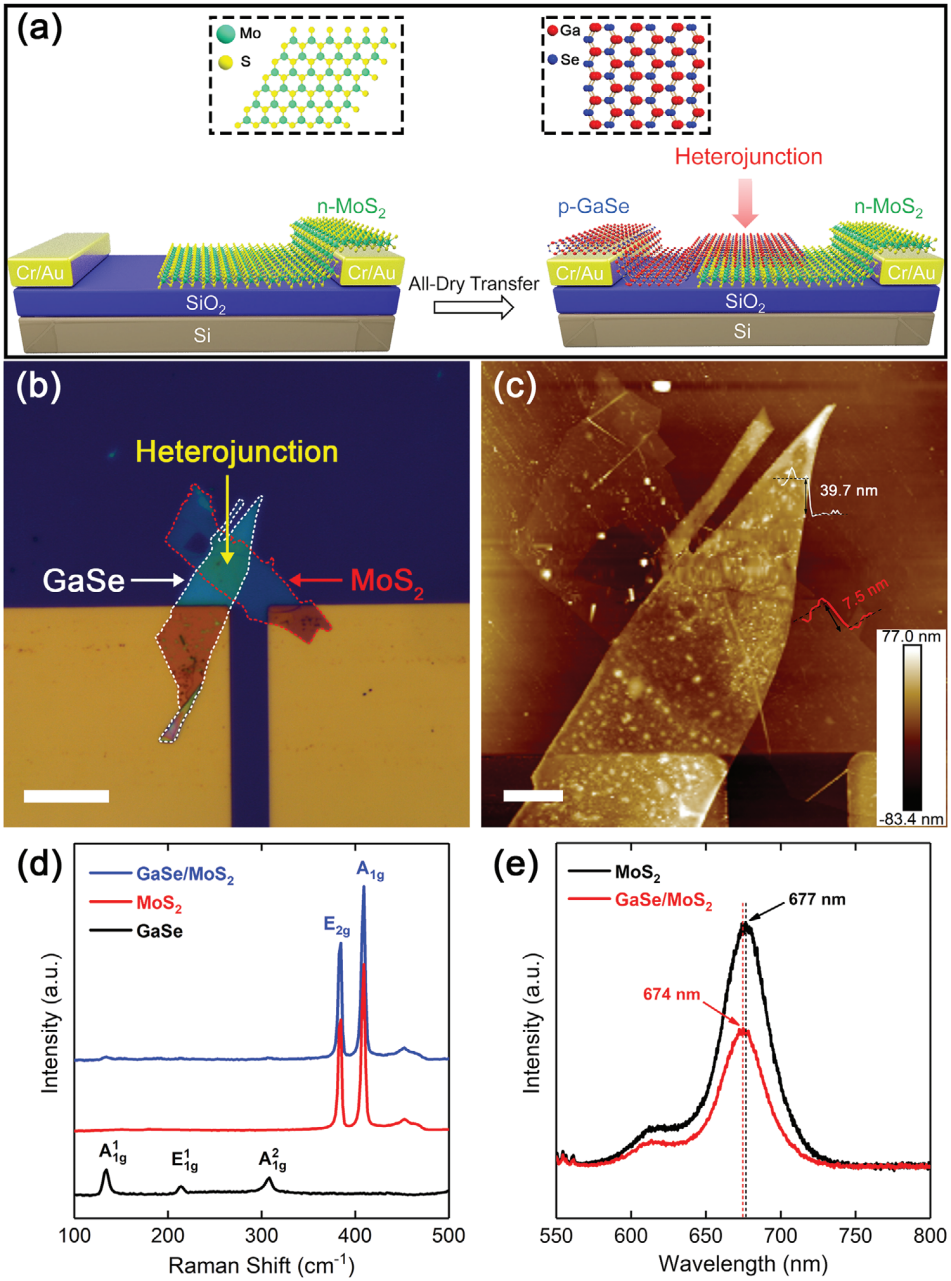
a) Schematic diagram of the fabrication of the MoS_2_/GaSe heterojunction. b) Optical image of the MoS_2_/GaSe heterojunction. Scale bar is 20 µm. c) AFM image and the corresponding height profiles of the heterojunction. Scale bar is 5 µm. d) Raman spectra for MoS_2_ and GaSe flakes and the overlapped region in the heterostructure under 532 nm laser. e) PL spectra of MoS_2_ and MoS_2_/GaSe heterojunction obtained with Raman spectrometer excited by a 532 nm laser. For GaSe, the intensity is too low to detect.

The vertically stacked MoS_2_/GaSe heterojunction was fabricated with all‐dry transfer method as illustrated in **Figure** **1**a. Briefly, the multilayer GaSe nanoflakes were mechanically exfoliated and transferred onto one side of the pre‐patterned Cr/Au electrodes.^[^ ^18^ ^]^ The multilayer MoS_2_ nanoflakes were then transferred onto the other side of electrodes and overlapped with the GaSe nanoflakes as shown in Figure 1b. The atomic force microscopy (AFM) image of the heterojunction is shown in Figure 1c. The height of the GaSe nanoflakes is ≈39.3 nm and that of the MoS_2_ nanoflakes is ≈7.5 nm. Then, the specific vibrational properties of the heterojunction were investigated by Raman spectroscopy. As shown in Figure 1d, the peaks of E_2g_ mode at 384.6 cm^−1^ and A_1g_ mode at 409.2 cm^−1^ are the fingerprint peaks of multilayer MoS_2_.^[^ ^19^ ^]^ Meanwhile, the peaks at 134.1, 213.9, and 308.3 cm^−1^ were the characteristic peaks of GaSe.^[^ ^20^ ^]^ The Raman spectra of the MoS_2_/GaSe heterojunction indicate the van der Waals feature of the heterojunction, among which the peak intensity of GaSe is relative lower. Figure 1e presents the photoluminescence (PL) spectra of MoS_2_ and MoS_2_/GaSe heterojunction. The intensive PL peak of MoS_2_ flakes was highly suppressed by GaSe in MoS_2_/GaSe heterojunction, which is attributable to the rapid separation of carriers. Compared with MoS_2_ flakes, the slight blue shift of the heterojunction is attributed to the p‐type doping of GaSe, indicating a strong interlayer coupling between MoS_2_ and GaSe.^[^ ^21^ ^]^

**Figure 2:**
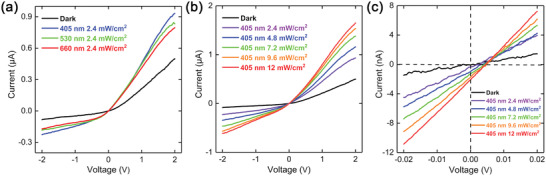
*I–V* curves of the MoS_2_/GaSe heterojunction. a) In darkness and under 2.4 mW cm^−2^ light illumination with different wavelengths. b) In darkness and under 405 nm light illumination with increasing power densities from 2.4 to 12 mW cm^−2^. c) Zoomed‐in plot of *I–V* characteristics of the MoS_2_/GaSe heterojunction.

The optoelectronic performances of the fabricated devices were characterized in darkness and upon illumination using a home‐made probe station system. **Figure** **2**a shows the *I–V* curves of the heterojunction in darkness and under 2.4 mW cm^−2^ light illumination with different wavelengths, which reveals a rectification behavior of the heterojunction. The photo‐response under 405 nm light illumination is higher than those under 530 and 660 nm light illumination, indicating the heterojunction is more sensitive to 405 nm light illumination. Then, we further tested the optoelectronic performance of the heterojunction under 405 nm light illumination with increasing power densities (Figure 2b). The photocurrent increases monotonically with the increasing illumination power density because of the photogeneration of electron–hole pairs that are separated by the applied drain–source bias voltage (photoconductive generation mechanism).^[^ ^22^ ^]^ Figure 2c shows the detailed *I–V* curves at low bias voltage. The built‐in electric field at the interface between MoS_2_ and GaSe separates the photogenerated electron–hole pairs at zero bias voltage, giving rise to the so‐called short‐circuit current (*I*_sc_), a fingerprint of the photovoltaic effect. *I*_sc_ shows a stable and repeatable response with light illumination switching (Figure S1, Supporting Information). The open‐circuit voltage (*V*_oc_) is also a signature of the built‐in electric field. Both *I*_sc_ and *V*_oc_ increase as the incident optical power increases (Figure S2, Supporting Information). What is more, we introduce a key parameter, Photoresponsivity (*R*), to evaluate the photosensitivity of the sensors and it is defined as Equation (1):

(1) $$R\;=I_{\mathrm{ph}}\;/P_{\mathrm{in}}$$

where *I*_ph_ is the photocurrent and *P*_in_ is the incident light power. At a 2.4 mW cm^−2^ illumination intensity and a voltage bias of 0.02 V, the heterojunction exhibits a responsivity of 841 mA W^−1^, which is comparable to black phosphorus/WSe_2_ heterojunction and one or two magnitudes larger than other reported heterojunction‐based photodetectors.^[^ ^23^ ^]^

**Figure 3:**
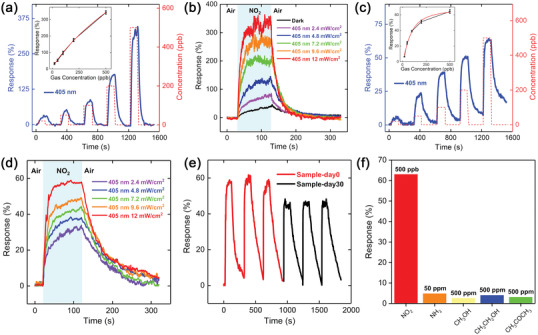
Gas sensing performance of the MoS_2_/GaSe heterojunction at room temperature. a) Dynamic response of the heterojunction exposed to different NO_2_ concentrations at 1 V bias voltage under 12 mW cm^−2^ 405 nm light illumination. b) Gas sensing response of the heterojunction toward 500 ppb NO_2_ under 405 nm light illumination with increasing power density. c) Self‐powered dynamic response of the heterojunction exposed to different NO_2_ concentrations under 12 mW cm^−2^ 405 nm light illumination. d) Self‐powered gas sensing response of the heterojunction exposed to 500 ppb NO_2_ under 405 nm light illumination with increasing power density. e) Self‐powered gas sensing stability of the heterojunction toward 500 ppb NO_2_ under 12 mW cm^−2^ 405 nm light illumination. Sample‐day0 (red curve) was tested right after sensor fabrication and Sample‐day30 (black curve) was tested after 30‐day exposure in ambient condition. f) The selectivity of the heterojunction to various gases under the same conditions.

The gas sensing experiments were performed by a home‐built gas sensing system.^[^ ^24^ ^]^ As it is discussed above, 405 nm light illumination was applied onto the sample to improve the gas sensing performance, since the heterojunction has best photo‐response to 405 nm light among the three wavelengths. By applying the visible light instead of UV light, the potential damage from the light source could be minimized and the illumination influence of the light sources and light intensities we applied on the temperature could be ignored.^[^ ^25^ ^]^ **Figure** **3**a shows the gas sensing performance of the heterojunction with various NO_2_ concentration under 12 mW cm^−2^ 405 nm light illumination (at 1 V bias voltage). The gas sensing response of the devices is defined as Equation (2):

(2) $$\mathrm{Response}\left(\%\right)\;=\frac{\left|R_{g}-R_{a}\right|}{R_{a}}\;\times \;100\%$$

where *R*_a_ is the resistance in air and *R*_g_ is the resistance in the NO_2_ atmosphere of the gas sensors. Taking advantage of the high surface‐to‐volume ratio, the MoS_2_/GaSe heterojunction shows a relatively high NO_2_ sensing response even at a very low concentration. The gas sensing response increases monotonically as the concentration rises from 20 to 500 ppb. Compared with other reported 2D materials‐based gas sensors, the heterojunction under illumination presents a higher response of 32% even toward 20 ppb NO_2_.^[^ ^11^, ^26^ ^]^ The data points in the inset of Figure 3a fit with Langmuir isotherm for molecules adsorbed on the surface with Equation (3):

(3) $$\mathrm{Response}\left(\%\right)=\frac{1998}{1+\frac{1000}{C{\left(\mathrm{ppb}\right)}^{0.853}}}\;\times \;100\%$$

where *C* is the concentration in ppb. The fitting curve confirms that the sensing mechanism for NO_2_ sensing is charge transfer between the absorbed gas molecules and the heterojunction.^[^ ^27^, ^28^ ^]^ Then we further explored the relationship between the gas sensing response and light power intensity. The gas sensing response under 405 nm light illumination with different power densities is shown in Figure 3b. The response toward 500 ppb NO_2_ monotonically rises from 44% to 346%, when the light power density increases from 0 to 12 mW cm^−2^. With the rise of the power density, more electrons are generated and transferred from the heterojunction to NO_2_, which accounts for the subsequent light enhancement of the gas sensing performance. Moreover, compared with the individual materials, the MoS_2_/GaSe heterojunction presented a higher gas sensing response under the same condition (Figure S4, Supporting Information). This phenomenon might be explained in terms of the high electron–hole pairs photo‐generated efficiency at the interface of the heterojunction under light illumination, which is consistent with the reported 2D material heterojunction‐based gas sensors.^[^ ^27^, ^29^ ^]^ Therefore, the function of the heterojunction is not only forming the p‐n junction to achieve the photovoltaic self‐powered gas sensing performance, but also facilitating the generation of photo‐induced carriers to improve the gas sensing properties of the gas sensor.

Based on the photovoltaic effect, the *I*_sc_ could be used as the signal to detect NO_2_ in the absence of external bias voltage.^[^ ^13^ ^]^ Accordingly, the photovoltaic self‐powered gas sensing experiments were conducted on MoS_2_/GaSe heterojunction under light illumination. The gas sensing response was calculated using the following Equation (4):

(4) $$\mathrm{Response}\left(\%\right)=\left|\frac{I_{g}-I_{a}}{I_{a}}\right|\;\times \;100\%$$

where *I*_a_ is the *I*_sc_ of the gas sensors in air and *I*_g_ is the *I*_sc_ in NO_2_ atmosphere of gas sensors. The corresponding dynamic response of *I*_sc_ with various NO_2_ concentration is shown in Figure 3c. The self‐powered gas sensing response increases from 6.3% (20 ppb) to 64.3% (500 ppb) and the heterojunction shows a good recovery at room temperature. The inset of Figure 3c shows that the gas sensing response also fits the Langmuir isotherm in Equation (5):

(5) $$\mathrm{Response}\left(\%\right)=\frac{68.72}{1+\frac{500}{C{\left(\mathrm{ppb}\right)}^{1.39}}}\;\times \;100\%$$

Since the carriers for self‐powered gas sensing at 0 V bias voltage all derive from the photogenerated carriers, the corresponding gas sensing response is lower than the above conventional response with bias voltage. However, it is worth mentioning that the LOD is the lowest among the reported photovoltaic self‐powered gas sensors so far.^[^ ^7^, ^9^, ^13^ ^]^ The self‐powered gas sensing performance with different light illumination power densities is illustrated in Figure 3d and the corresponding response of *I*_sc_ is shown in Figure S5, Supporting Information. The same variance tendency of gas sensing response identifies with the one with bias voltage, representing decent photo‐response and gas sensing performance. To evaluate the noise immunity of the self‐powered gas sensing properties, the signal‐to‐noise‐ratio (SNR) and the theoretical LOD of the heterojunction were calculated with the root‐mean square current noise amplitude (the detailed calculation processes see Supporting Information).^[^ ^30^ ^]^ The SNR of the photovoltaic self‐powered gas sensing at 500 ppb NO_2_ is 54524.83, which is quite comparable with the reported metal oxide‐based gas sensors by applying bias voltage at the same gas concentration level.^[^ ^31^ ^]^ The response time was measured from the injection of the gases to 90% of an end point (maximum), and the recovery time was measured at the point where the current hits the one‐tenth point near the minimum from the maximum. According to the transient response characteristic of the heterojunction toward 500 ppb under 12 mW cm^−2^ 405 nm light illumination (Figure S6, Supporting Information), the response and recovery time is respectively 23 and 178 s, which is one of the top three shortest response and recovery time toward ppb‐level NO_2_ at room temperature among the reported 2D material‐based gas sensors so far.^[^ ^24^, ^32^ ^]^

The self‐powered gas sensing stability of the MoS_2_/GaSe heterojunction is demonstrated in Figure 3e. The heterojunction still exhibits an average response of 46% toward 500 ppb NO_2_ after 30‐day exposure in ambient atmosphere, showing about 23% decay relative to the initial value, which reveals that the heterojunction possesses relatively long‐term stability, and the gas sensing performance reduction might be caused by the exposed part of GaSe in the heterojunction. To investigate the humidity effect on the gas sensing performance, the MoS_2_/GaSe heterojunction was exposed to different relative humidity (RH) from 20% to 70% under 12 mW cm^−2^ 405 nm light illumination and the corresponding gas sensing response toward 500 ppb NO_2_ was recorded (Figure S7, Supporting Information). We found that the gas sensing performance of MoS_2_/GaSe heterojunction would be suppressed along with the increase of RH. The same phenomena have also been reported from other 2D material‐based gas sensors.^[^ ^26^, ^33^ ^]^ Moreover, the sensor shows an outstanding selectivity toward NO_2_ among other interfering gases including NH_3_, methanol, ethanol, and acetone as shown in Figure 3f.

**Figure 4:**
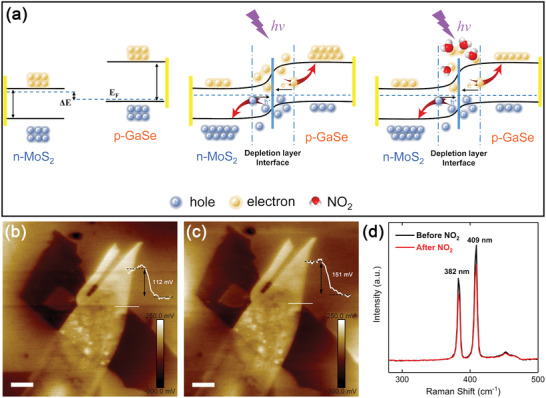
a) Schematic diagram of the sensing mechanism of the heterojunction. Band diagram of isolated MoS_2_ and GaSe (left), heterojunction between the two materials under light illumination (middle) and the heterojunction with NO_2_ exposure (right). KPFM images of the heterojunction b) before and c) after exposing to NO_2_. Scale bars are 5 µm. d) Raman spectra of the heterojunction before and after NO_2_ exposure at room temperature.

The mechanism of photovoltaic self‐powered gas sensing can be explained by the band diagrams of MoS_2_/GaSe heterojunction schematically depicted in **Figure** **4**a. The isolated materials (left panel) are semiconductors with bandgap energies of 1.30 and 2.05 eV, respectively, for multiple‐layered MoS_2_ and GaSe.^[^ ^34^, ^35^ ^]^ When the two isolated materials are brought together, the difference in the position of the Fermi levels creates a built‐in electric field, which induces the bending of both the conduction and the valence band at the interface (middle panel). When the p‐n junction is exposed to light illumination, electron–hole pairs in both MoS_2_ and GaSe layers are photogenerated and tend to migrate at the interface. Owing to the build‐in electric field, the electrons are transferred to n‐type MoS_2_ and the holes to p‐type GaSe, leading to the separation of carriers. At zero applied bias, the photogenerated electron–hole pairs produce an *I*_sc_. The concentration of electrons would decrease since the NO_2_ has a strong electron affinity and can easily capture electrons from the conduction band of the materials (right panel). As a result, the resistance of the heterojunction dramatically increases and the *I*_sc_ decreases upon NO_2_ exposure. To experimentally investigate the charge transfer and band shift process, Kelvin potential force microscopy (KPFM) was adopted before and after NO_2_ exposure. The contact potential difference, as known as surface potential, is determined by the work function difference between the sample (*W*_sample_) and the AFM tip (*W*_tip_) (Equation (6)):

(6) $$\mathrm{CPD}=\left(W_{\mathrm{sample}}-W_{\mathrm{tip}}\right)/e$$

where *e* is the elementary charge.^[^ ^36^ ^]^ In our case, surface potential of the heterojunction were obtained before and after exposing to NO_2_. The surface potential profiles were measured along the white lines and the surface potential of SiO_2_ substrate was set as reference. As shown in Figure 4b,c, after NO_2_ exposure, the surface potential of the heterojunction increases to 151 from 112 mV, indicating the Fermi level is shifted toward the valence band.^[^ ^37^ ^]^ The results indicate that the electrons are transferred from the heterojunction to NO_2_ molecules. The same phenomena were also observed in other 2D materials‐based heterojunctions.^[^ ^27^, ^38^ ^]^

To investigate the NO_2_ sensing mechanism of the MoS_2_/GaSe heterojunction, Raman spectra were adopted before and after NO_2_ exposure at room temperature. The magnified images of the in‐plane (E_2g_) and out‐of‐plane (A_1g_) vibrational modes of MoS_2_ are shown in Figure 4d. The peak positions remain the same, suggesting the structure of the heterojunction is stable after NO_2_ exposure. However, the intensities of E_2g_ and A_1g_ exhibited distinct decrease after exposing to NO_2_, which is caused by the phonon self‐energy renormalization and weakening of the phonons. This phenomenon indicates that NO_2_ is physically absorbed on the surface of MoS_2_/GaSe junction.^[^ ^39^ ^]^ A similar behavior has been reported in the case of MnPS_3_.^[^ ^28^ ^]^

We have summarized recent works on various self‐powered gas sensors in **Table** **1**. Compared with the self‐powered gas sensors based on piezoelectric or triboelectric theory, the photovoltaic based sensors possess lower responses but with no need for applying extra mechanical energy. The gas sensing response of the obtained MoS_2_/GaSe heterojunction is quite comparable to the listed photovoltaic based gas sensors toward low concentration NO_2_. It is noteworthy that the LOD toward NO_2_ of our devices is the lowest among the reported self‐powered gas sensors, which might be owing to the excellent intrinsic gas sensing properties of MoS_2_ and GaSe.^[^ ^16^, ^47^ ^]^

**Table 1:** Comparison of room‐temperature self‐powered gas sensing performance of our gas sensor with the reported self‐powered gas sensors

Materials	Mechanism	Target gas	Conc.^a)^ [ppm]	Response^b)^ [%]	Light wavelength	Ref.
ZnO	Piezoelectric	H_2_S	100	13.1^A^	No	^[^ ^40^ ^]^
NiO/ZnO	Piezoelectric	H_2_S	100	31.5^A^	No	^[^ ^41^ ^]^
Pd/ZnO	Piezoelectric	Ethanol	200	15.6^A^	No	^[^ ^42^ ^]^
SnO_2_/ZnO	Piezoelectric	H_2_	200	22.8^A^	No	^[^ ^43^ ^]^
NiO/ZnO (polyimide)	Triboelectric	Ethanol	10^3^	≈37.5^A^	No	^[^ ^6^ ^]^
PANI/PTFE/PANI	Triboelectric	Ethanol	210	66.8^B^	No	^[^ ^44^ ^]^
Pd‐ITO (PET)	Triboelectric	H_2_	10^4^	75^A^	No	^[^ ^45^ ^]^
ZnO‐RGO	Triboelectric	NO_2_	100	≈1680^A^	365 nm (40 mW cm^−2^)	^[^ ^46^ ^]^
p‐SWNT/n‐Si	Photovoltatic	H_2_S	0.4	2.23^A^	600 nm (1.8 mW cm^−2^)	^[^ ^7^ ^]^
CdS@n‐ZnO/p‐Si	Photovoltatic	Ethanol	200	≈6.5^A^	Solar (100 mW cm^−2^)	^[^ ^8^ ^]^
p‐Si/n‐ZnO	Photovoltaic	NO_2_	0.75	23.5 (Amine)^A^ 12.8 (Thiol)^A^	Solar (30 mW cm^−2^)	^[^ ^9^ ^]^
p‐SiNWs/ITO	Photovoltatic	NO_2_	0.5	≈87.5^B^	576 nm (20 mW cm^−2^)	^[^ ^14^ ^]^
WSe_2_/WS_2_	Photovoltatic	NO_2_/NH_3_	500	322/23^B^ (Self‐powered mode)	Solar (10 W/m^2^)	^[^ ^13^ ^]^
MoS_2_/WSe_2_				36/165^B^ (Self‐powered mode)		
MoS_2_/GaSe	Photovoltatic	NO_2_	0.02	6.3^B^ (Self‐powered mode)	405 nm (12 mW cm^−2^)	This work
				32.2^B^ (1 V bias voltage)		

^a)^ Conc. represents concentration

^b)^ For convenience of comparison, the evaluation of the response is converted as A: $\mathrm{Response}\;\left(\%\right)\;=|\frac{V_{g}-V_{a}}{V_{a}}|\;\times \;100\;\%$ and B: $\mathrm{Repsonse}\;\left(\%\right)=|\frac{I_{g}-I_{a}}{I_{a}}|\;\times \;100\%$

## 3 Conclusion

In summary, the MoS_2_/GaSe heterojunction‐based photovoltaic self‐powered gas sensors were fabricated by mechanical exfoliation and all‐dry transfer method. Our device presented a relative high response and superior reversibility and selectivity at room temperature under 405 nm light illumination both with bias voltage and in self‐powered mode. The separation of photogenerated carriers with the built‐in electric field acted as the driving forces, indicating a photovoltaic self‐powered gas sensing behavior. Importantly, the LOD of our device (20 ppb) toward NO_2_ is the lowest of the reported self‐powered room‐temperature gas sensors so far. Moreover, by KPFM and Raman spectra, the electron transfer between heterojunction and NO_2_ molecules was confirmed to be the dominant mechanism for NO_2_ sensing. The MoS_2_/GaSe heterojunction with excellent gas sensing performances could be regarded as a promising sensing platform for low power consumption environmental monitoring systems.

## 4 Experimental Section

### Preparation of MoS_2_ and GaSe Nanosheets

Through mechanical exfoliation method, MoS_2_ nanosheets were prepared from the bulk MoS_2_ crystals (SixCarbon Technology Shenzhen, China). First, the MoS_2_ crystal was covered by a piece of Nitto tape (Nitto Denko, Japan, SPV 224P) followed by scrupulously peeling off the tape. Then a poly(dimethylsiloxane) (PDMS) viscoelastic stamp (Gel‐pak, USA, WF‐30‐X4) was pasted on the MoS_2_ sheets. Eventually, the MoS_2_ nanosheets could be obtained on the PDMS stamp by slowly removing the stamp off. The GaSe nanosheets were prepared with the same approach.

### Fabrication of MoS_2_/GaSe Heterojunction‐Based Gas Sensors

The multilayer MoS_2_ nanosheet was inspected and selected with polarization microscope (Leica Microsystems, Germany, DM2700P) and then deterministically transferred from the PDMS stamp to one side of the Cr/Au electrodes pre‐patterned on the SiO_2_/Si wafer by all‐dry transfer method. The multilayer GaSe nanosheet was then transferred onto the other side of Cr/Au electrodes and overlapped with the MoS_2_ nanosheet to fabricate MoS_2_/GaSe heterojunction‐based gas sensor.

### Characterization

The optical image of MoS_2_/GaSe heterojunction was obtained on DM2700P polarization microscope with reflection mode. The AFM and KPFM images were acquired by atomic force microscope (Bruker, USA, Multimode 8). The Raman spectra and PL spectra were tested by Raman microscope (RENISHAW, UK, inVia) under the 532 nm excitation wavelength before and after injecting the NO_2_ gas in a quartz chamber.

### Photoelectric and Gas Sensing Properties Tests

The photoelectric properties tests of the MoS_2_/GaSe heterojunction in darkness and under light illumination were performed though a Keithley 2450 source meter (Tektronix, USA). The power density of the light‐emitting diode (LED) light sources was modulated by a power supplier and calibrated by a silicon photodiode sensor (Thorlabs, USA, S120VC). All the gas sensing tests were performed in a home‐built gas sensing system. For the photovoltaic self‐powered gas sensing tests, the LED light sources were assembled with the gas sensing chamber through a standard optical fiber at dark room. The details of gas sensing tests are shown in the Supporting Information.

## Conflict of Interest

The authors declare no conflict of interest.

## Supporting information

Supporting Information

## Data Availability

Research data are not shared.
