# Supplementary material for: A Photovoltaic Self‐Powered Gas Sensor Based on All‐Dry Transferred MoS2/GaSe Heterojunction for ppb‐Level NO2 Sensing at Room Temperature
Source: Adv Sci (Weinh). 2021 May 24;8(14):2100472. doi: 10.1002/advs.202100472 (PMC8292907; doi:10.1002/advs.202100472)
Supplement: Supplementary file 1 — Supporting Information [file ADVS-8-2100472-s001.pdf]

© 2021 Wiley-VCH GmbH

ADVANCED SCIENCE
Open Access

Supporting Information

for *Adv. Sci.*, DOI: 10.1002/advs.202100472

A Photovoltaic Self-Powered Gas Sensor Based on All-Dry Transferred MoS_2/GaSe Heterojunction for ppb-Level NO_2 Sensing at Room Temperature

Yue Niu, Junwei Zeng, Xiangcheng Liu, Jialong Li, Quan Wang, Hao Li, Nicolaas Frans de Rooij, Yao Wang* and Guofu Zhou*

Supporting Information

A Photovoltaic Self-Powered Gas Sensor Based on All-Dry Transferred MoS_2/GaSe Heterojunction for ppb-Level NO_2 Sensing at Room Temperature

Yue Niu[1, 2, ‡, *], *Junwei Zeng*[1, 2, ‡], *Xiangcheng Liu*[1, 2], *Jialong Li*[1, 2], *Quan Wang*[1, 2], *Hao Li*[1, 2], *Nicolaas Frans de Rooij*[1, 2], *Yao Wang*[1, 2, *] and *Guofu Zhou*[1, 2]

[1]Guangdong Provincial Key Laboratory of Optical Information Materials and Technology, Institute of Electronic Paper Displays, South China Academy of Advanced Optoelectronics, South China Normal University, Guangzhou, 510006,P. R. China

[2]National Center for International Research on Green Optoelectronics, South China Normal University, Guangzhou, 510006, P. R. China

[‡]*These authors contributed equally tothis work.*

[*]*Corresponding Author: Yao Wang, Email: wangyao@m.scnu.edu.cn*

Yue Niu, Email: niuyue@m.scnu.edu.cn

1. Gas sensing test process.

In our gas sensing test process, the desired NO_2 concentrations of 20, 50, 100 ,200 and 500 ppb were obtained by injecting 3.4, 8.5, 17, 34 and 85 mL of 100 ppm NO_2 respectively into the gas sensing chamber (the volume of the chamber is 17 L). For NH_3, the concentration of 50 ppm was obtained by injecting 85 mL 10000 ppm NH_3 into the gas sensing chamber. For volatile organic compounds (VOCs, including methanol, ethanol and acetone), their vapor steam can be generated by heating the corresponding liquid in a heater inside the chamber. The desired concentration (C) was calculated by the following formula:

$$C = \frac{22.4 \times \rho \times \varphi \times V_1}{M \times V_2} \times 1000$$

where C (ppm)is the target gas concentration, ρ (g/mL) is the density of the VOCs liquid, φ is the required gas volume fraction, V_1 (μL) is the volume of the required VOCs liquid and V_2 is the volume of the gas sensing chamber, respectively. After the target gases were injected into the chamber for 100 s, the chamber cover was opened to let the gas sensors recover in the atmosphere. Then the conductance of the sensor will recover to its initial value and another response process can be conducted.All the gas sensing tests were carried out in the same environment at room temperature (25 °C) and a certain relative humidity range (20%-70%), which were controlled by an air conditioner.

2. calculation of SNR value and theoretical LOD

To analyze whether the SNR of gas sensing processes influences the response or not, we calculated the SNR value and theoretical limit of detection (LOD) of the self-powered gas sensor,[1] and the detailed calculation processes are shown below:

Step 1: Take 10 data points at the baseline (self-powered mode) before the NO_2 exposure:

Time (s)	Y_i ($\Delta R/R$, %)
2.09033	0.00681
3.99889	0.00446
6.08925	0.00651
7.99779	0.00559
9.99719	0.00989
11.99675	0.00552
14.08708	0.00576
15.99566	0.00607
17.99510	0.00589
19.99449	0.00817

Step 2: Plot the data (Y_i versus time) and then execute a fifth order polynomial fit within the data point range. The fifth-order polynomial fit gives not only the curve-fitting equation but also the statistical parameters of the polynomial fit:

6

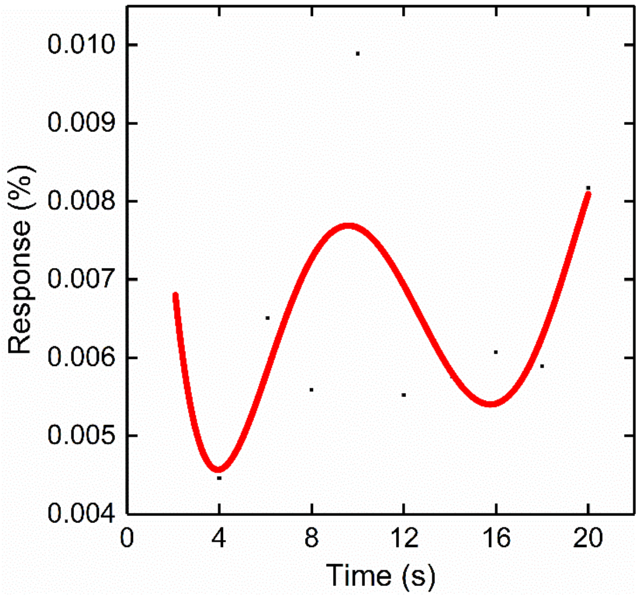

Curve-fitting equation obtained from the fifth order polynomial fit:

$$Y = 0.01923-0.00987*X+0.00231*X^2-2.26081*10^{-4}*X^3+9.75849*10^{-6}*X^4-1.53219*10^{-7}*X^5$$

Step 3:

$$V_{X^2} = \Sigma(Y_i - Y)^2$$

where Y_i is the measured data point and Y is the corresponding value calculated from the above curve-fitting equation. The values of Y_i and Y are tabulated below:

Time (s)	Y_i (ΔR/R, %)	Y
1.999460	0.00681	0.006807193
4.089790	0.00446	0.004582027
6.089250	0.00651	0.005869984
8.088690	0.00559	0.007305451
9.906310	0.00989	0.007712340
11.90586	0.00552	0.006990019
13.90530	0.00576	0.005882797
15.90478	0.00607	0.005512445
18.08599	0.00589	0.006391396
19.90360	0.00817	0.008234477

Step 4: The V_{X^2} value is then calculated as shown below:

$$V_{X^2} = \Sigma(Y_i - Y)^2$$

Time (s)	Y_i (ΔR/R, %)	Y	$(Y_i-Y)^2$
1.999460	0.00681	0.006807193	7.88169E-12
4.089790	0.00446	0.004582027	1.48906E-08
6.089250	0.00651	0.005869984	4.09621E-07
8.088690	0.00559	0.007305451	2.94277E-06
9.906310	0.00989	0.007712340	4.7422E-06
11.90586	0.00552	0.006990019	2.16096E-06
13.90530	0.00576	0.005882797	1.50791E-08

15.90478	0.00607	0.005512445	3.10868E-07
18.08599	0.00589	0.006391396	2.51398E-07
19.90360	0.00817	0.008234477	4.15727E-09
		V_{X^2}	**1.0852E-05**

Step 5: The root-mean-square I_{sc} noise (rms_{noise}) is defined as:

$$rms_{noise} = \sqrt{\frac{V_{X^2}}{N}}$$

where N = 10 (10 data points were used in the curve fitting). rms_{noise} = 0.001041727.

Step 6: the SNR of the gas sensor in self-powered mode can be expressed as:

$$SNR = \frac{\Delta I}{rms_{noise}} = \frac{|I_g - I_a|}{\sqrt{\frac{V_{X^2}}{N}}}$$

According tothe calculation, the SNR = 54524.83

According to the IUPAC definition,[2] a true signal must be at least 3 times larger than SNR. Therefore, the theoretical LOD can be extrapolated from the linear calibration curve when the signal equals 3 times the noise:

$$LOD\ (ppb) = 3 \times \frac{rms_{noise}}{slope}$$

where slope obtained from linear calibration curve of response versusconcentration plot (Figure 3c inset). For our MoS_2/GaSe heterojunction, the theoretical LOD is 0.0312 ppb (31.2 ppt). In our case, the experimental LOD of the MoS_2/GaSe heterojunction is 20 ppb, which is much higher than the theoretical one, indicating that the SNR fluctuation will not affect the self-powered gas sensing of MoS_2/GaSe heterojunction.

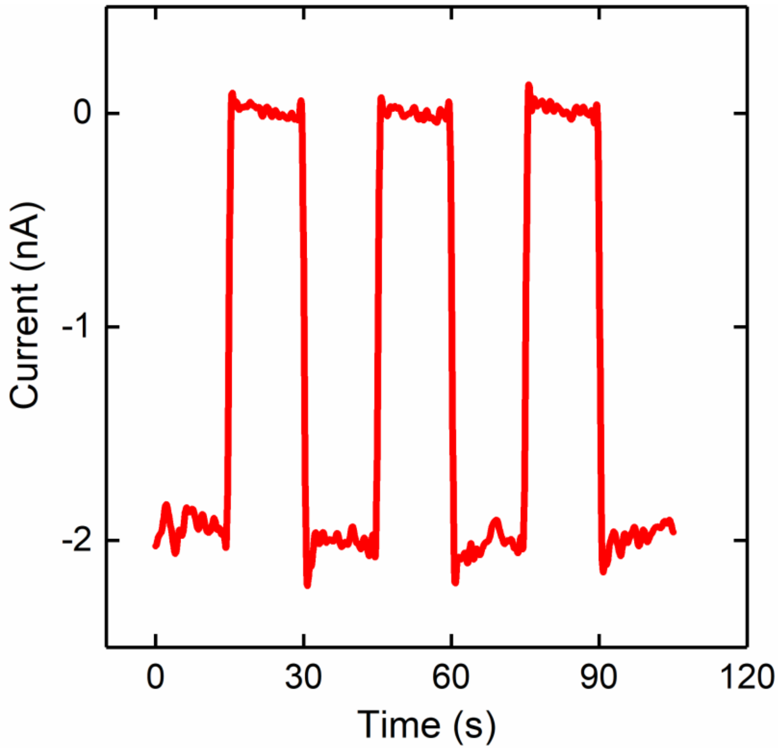

Figure S1. Time-dependent photocurrent of MoS_2/GaSe heterojunction excited by 12 mW/cm^2 405 nm light illumination.

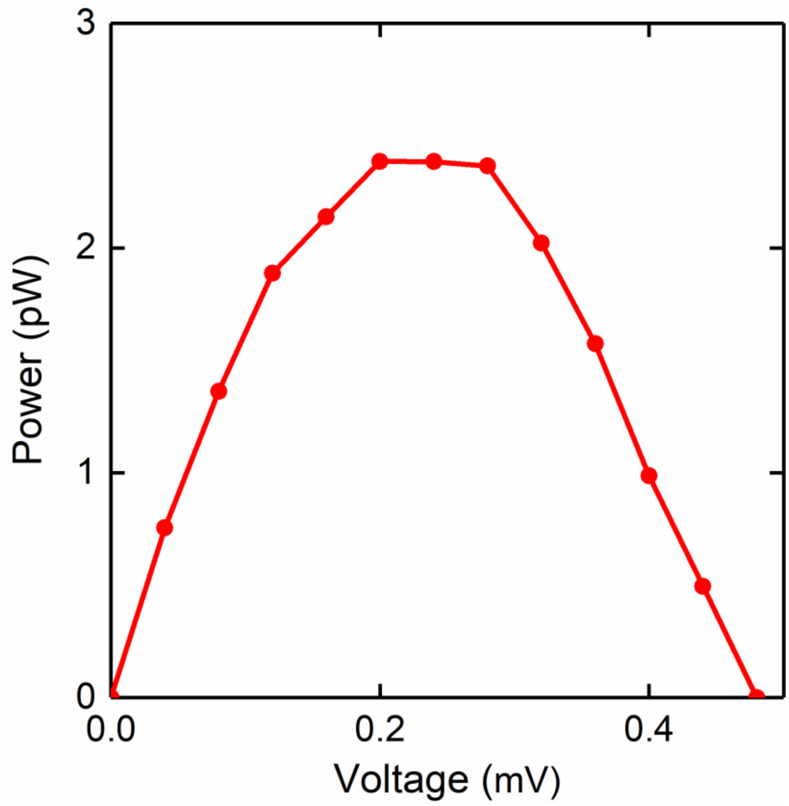

Figure S2. Generated power fromthe heterojunction under 405nm light illumination(power density: 12 mW/m^2) as a function of voltage with current.

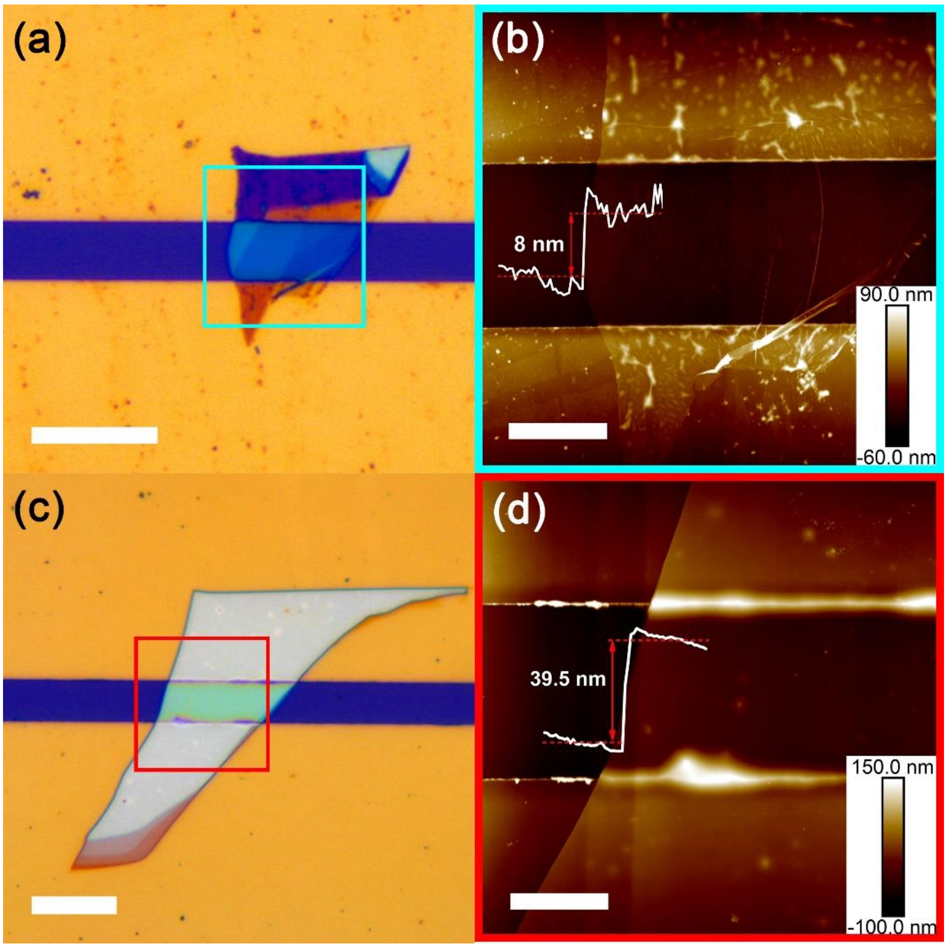

Figure S3. The optical images of (a) individual MoS_2 and (c) GaSe. The scale bars are 20 μm.

The AFM images of (b) individual MoS2 and (d) GaSe. The scale bars are 6 μm.

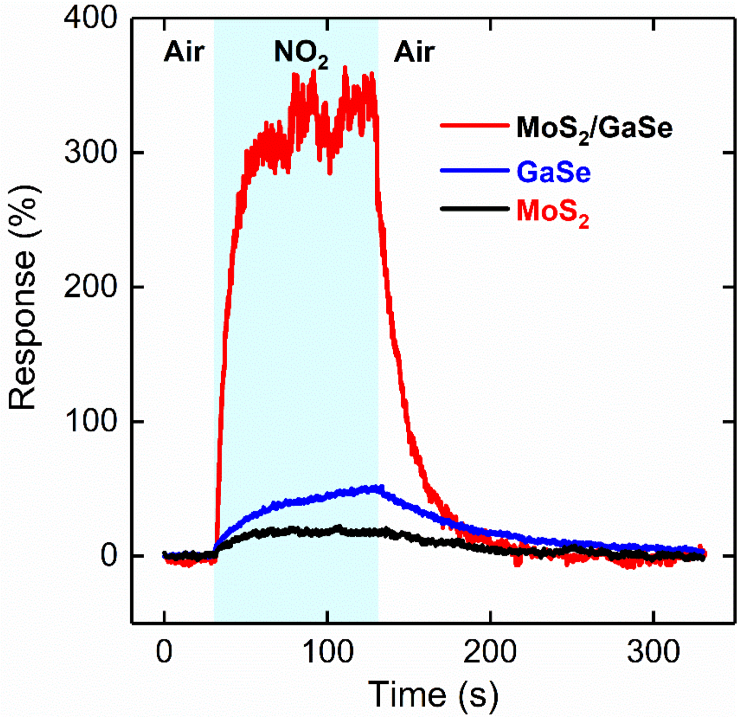

Figure S4. The gas sensing responses comparisons of the MoS_2/GaSe heterojunction (red), individual GaSe (blue) and individual MoS_2 (black) toward 500 ppb NO_2 under 12 mW/cm^2 405 nm light illumination at room temperature.

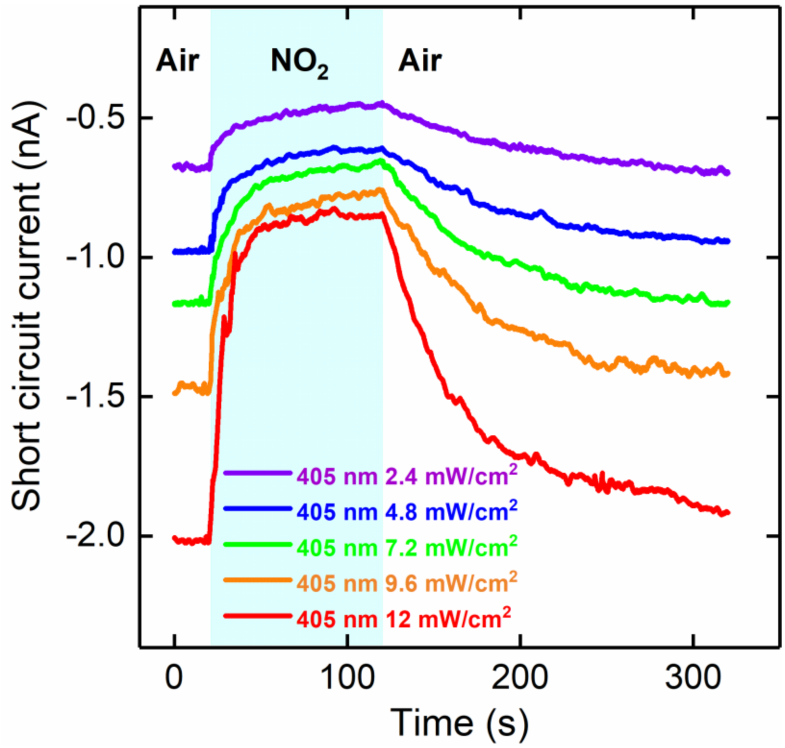

Figure S5. Transient response self-powered gas sensing characteristic of the heterojunction toward 500 ppb NO_2 under 405 nm light illumination with the power density of 12 mW/cm^2.

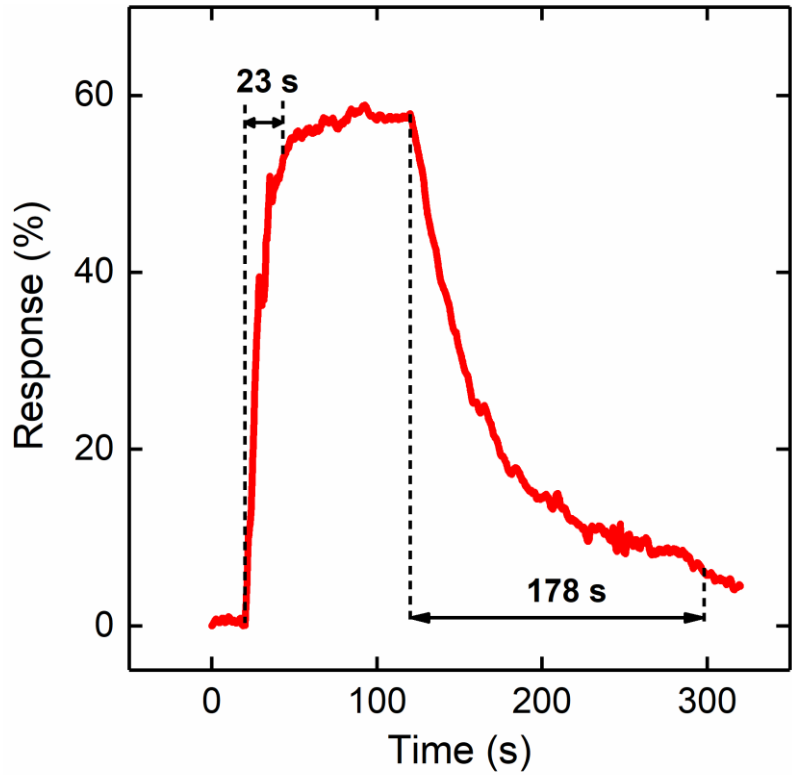

Figure S6. The transient response characteristic of the heterojunction toward 500 ppb under 12 mW/cm^2 405 nm light illumination.

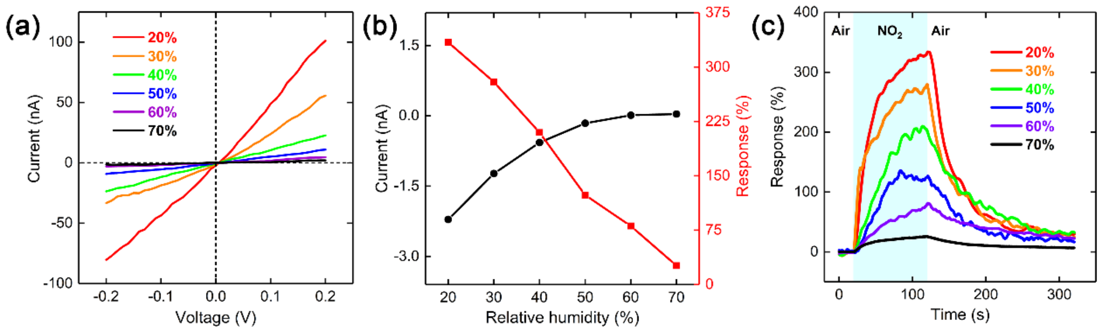

Figure S7.(a) *I-V* curves of MoS_2/GaSe heterojunctionin moisture atmosphere (RH ranged from 20% to 70%) under 12 mW/cm^2 405 nm light illumination. (b) The variation tendencies of I_{sc} (black) and gas sensing response of MoS_2/GaSe heterojunction toward 500 ppb NO_2 in moisture atmosphere (RH ranged from 20% to 70%) under 12 mW/cm^2 405 nm light illumination (red). (c) The gas sensing performances of MoS_2/GaSe heterojunction toward 500 ppb NO_2 in moisture atmosphere (RH ranged from 20% to 70%) under 12 mW/cm^2 405 nm light illumination.

In details, as shown in Figure S7a, the result showed a distinct decrease in the slope of *I-V* curves with an increase of RH. Water vapor is considered to be an electron acceptor just like NO_2, so the resistance of gas sensor increases along with the RH value rises,[3] resulting the absolute value of short circuit current ($|I_{sc}|$) decreases subsequently (Figure S7b). Since $|I_{sc}|$ dramatically decreases with the rise of RH, the SNR could not be ignored at high RH. We hence tested the traditional gas sensing performance of the MoS_2/GaSe heterojunction instead of the self-powered gas sensing performance in moisture atmosphere. As shown in Figure S7b and Figure S7c, the gas sensing responses also attenuated with the increasement of RH.

Reference

[1] V. Dua, S. P. Surwade, S. Ammu, X. Zhang, S. Jain, S. K. Manohar, Macromolecules 2009, 42, 5414.

[2] L. A. Currie, Pure and applied chemistry 1995, 67, 1699.

[3] D. J. Late, Y.-K. Huang, B. Liu, J. Acharya, S. N. Shirodkar, J. Luo, A. Yan, D. Charles, U. V. Waghmare, V. P. Dravid, C. N. R. Rao, ACS Nano 2013, 7, 4879.
